# Human mini-blood–brain barrier models for biomedical neuroscience research: a review

**DOI:** 10.1186/s40824-022-00332-z

**Published:** 2022-12-16

**Authors:** Minh Tran, Chaejeong Heo, Luke P. Lee, Hansang Cho

**Affiliations:** 1 Institute of Quantum Biophysics, Sungkyunkwan University, Suwon, 16419 Korea; 2 Department of Biophysics, Sungkyunkwan University, Suwon, 16419 Korea; 3 Department of Intelligent Precision Healthcare Convergence, Sungkyunkwan University, Suwon, 16419 Korea; 4 Center for Integrated Nanostructure Physics, Institute for Basic Science, Suwon, 16419 Korea; 5 Department of Bioengineering, University of California at Berkeley, Berkeley, CA USA; 6 Department of Electrical Engineering and Computer Science, University of California at Berkeley, Berkeley, CA USA; 7 Department of Medicine, Harvard Medical School, Brigham and Women’s Hospital, Boston, MA 02115 USA

**Keywords:** Blood–brain barrier, Human mini-brains, Neurological disorder, Personalized medicine

## Abstract

The human blood–brain barrier (BBB) is a unique multicellular structure that is in critical demand for fundamental neuroscience studies and therapeutic evaluation. Despite substantial achievements in creating in vitro human BBB platforms, challenges in generating specifics of physiopathological relevance are viewed as impediments to the establishment of in vitro models. In this review, we provide insight into the development and deployment of in vitro BBB models that allow investigation of the physiology and pathology of neurological therapeutic avenues. First, we highlight the critical components, including cell sources, biomaterial glue collections, and engineering techniques to reconstruct a miniaturized human BBB. Second, we describe recent breakthroughs in human mini-BBBs for investigating biological mechanisms in neurology. Finally, we discuss the application of human mini-BBBs to medical approaches. This review provides strategies for understanding neurological diseases, a validation model for drug discovery, and a potential approach for generating personalized medicine.

## Introduction

The blood–brain barrier (BBB) serves a crucial function in maintaining brain homeostasis by acting as a selective barrier that allows nourishment delivery while restricting the circulation of hazardous substances [1–3]. Owing to their unique structure, neurological drugs with enhanced BBB penetration are gaining popularity [4–6]. Additionally, recent clinical manifestations of neurodegeneration and cognitive decline are associated with vascular dysfunction, particularly BBB failure [5, 7, 8]. Regrettably, the mechanism linking BBB failure to neurodegenerative diseases remains unclear despite various experiments showing physiological and pathological processes. Therefore, an artificial framework for BBB culture systems has been constructed to increase our understanding of BBB physiopathology and the prerequisites for neuronal drug development. Indeed, artificial BBB models must recapitulate the dynamic, multi-component nature of neurovascular units (NVUs) and their unique anatomical and physiological characteristics, which are crucial for BBB physiopathogenesis and treatment response.

In artificial circumstances, by delivering certain cell types and biomaterial glues using microengineering technologies, the features of the in vivo BBB properties can be replicated [9, 10]. Traditionally, single or multiple cells are seeded into the extracellular matrix (ECM), where they exhibit BBB capability [10]. Numerous cellular programming and biomaterial engineering strategies have been developed to improve BBB formation under specific in vitro settings [11–13]. Since then, human in vitro BBB models has gradually evolved from simple two-dimensional (2D) cultures to sophisticated three-dimensional (3D) models in structural assembly. The concept of 3D BBB culture assists in making in vitro BBB models more representative of genuine BBB physiological properties [14]. Compared to conventional 2D BBB culture, 3D BBB models generate more consistent levels of gene and protein expression [10, 14]. Additionally, the extraordinary structural design combines advances in cellular reprogramming and biomaterial glues to enable recapitulation of the native BBB model, which serves as a human mini-BBB model. In the future, in vitro human mini-BBB models may eventually serve as a platform to replace animal trials and develop next-generation medicine, which is essential for personalized therapy. Numerous in vitro BBB-related advances have been reported in the fundamental research and biomedical fields of neurology. Nonetheless, in vitro BBB models have several limitations that must be addressed. These include anatomical structures and physiological functions that do not adequately imitate the complexity of native BBB function, the use of fluid flow as mechanical stimulation demands technical skill, and the limited period of the culture system results in genotype and phenotype failure. These disadvantages have encouraged the development of various techniques in cell programming, biomaterial engineering, and microfabrication to establish equivalence to in vivo models.

In this review, as an overview of the process of developing an in vitro human BBB model, we begin with a description of the biological features of the BBB, including its physiological and pathological aspects, with an emphasis on the metabolite transport route across the BBB. Then, we present a state-of-the-art way to replicate the in vitro human BBB model by describing various bio-components and engineering technologies, which provides a brief overview of the micromanufacturing format. Following that, we investigate the biological mechanism and their potential application in developing new medicines for neurological disorders. Specifically, using patient samples with contemporary methodologies reveals the ability to replicate real patient reactions relevant to neurological pathogenesis research for enhanced drug development and personalized medicine. Finally, we will discuss the difficulties and possibilities of evaluating of the respective advantages and disadvantages of the field.

## Physiopathological properties of the BBB

### Organization of the BBB layers

**Fig. 1:**
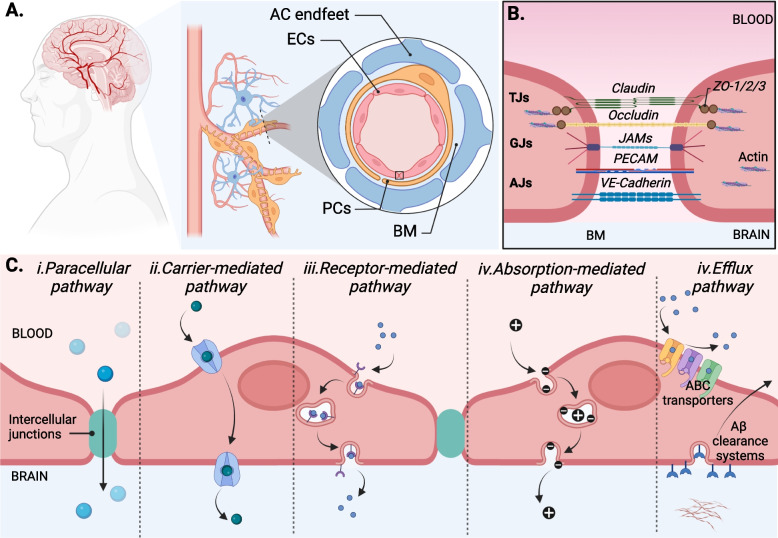
Physiological properties of BBB. **A**. The BBB structure. Schematic diagram of the endothelial cells, pericytes, astrocytes, and basement membrane forming a lumen of brain capillary structures. **B**. Junctional complexes structure. Single endothelial cell connects by tight junctions (Claudin, Occludin, ZO-1/2/3), gap junctions (JAMs), and adherens junctions (PECAM, VE-cadherin). **C**. Several different routes of transport across the BBB. i. Paracellular diffusion of gas, water, and small molecules. Transcellular diffusion of high lipid solubility molecules, ii. active transport via carriers of nutrient transport such as glucose, peptide, fatty acid, and nucleic acid, iii. Receptor-mediated transcytosis pathway requires the binding of a receptor on membranous vesicular trafficking. iv. Absorption-mediated transcytosis involves transport through membranous vesicular trafficking via positively charged molecular carriers. v. Active efflux pump systems include ABC transporters, including P-gp, BCRP, and MRP4 inhibit the new metabolites into the brain, whereas Aβ clearance systems reintroduce endogenous metabolites into the bloodstream. ECs represents endothelial cells; PC, pericytes; ACs, astrocytes; BM, basement membrane; TJs, tight junction; GJs, gap junctions; AJs, adherens junction

The central nervous system (CNS) plays a critical role in regulating survival, necessitating strict protection. Under physiological conditions, the BBB regulates the transformation of substances in the blood to protect the CNS from toxins and pathogens [15]. In the cerebrovascular structure, at the interior of the blood surface, mature endothelial cells (ECs) develop continuous junctional complexes to form the most significant interface between the blood and the CNS [Fig. 1A]. At each intercellular cleft between cells, a variety of transmembrane proteins, including tight/occluding junctions (e.g., occludin and claudin), adherent junctions (e.g., cadherin), and gap/communicating junctions (e.g., connexin), form a significant physical barrier that limits molecule transport [16] [Fig. 1B]. In addition, luminal EC surfaces contain mechanosensor systems that can sense shear stress from blood flow and turn it into a biochemical signal for the alteration of proteome and transcriptome patterns associated with health and disease [17, 18]. In contrast, abluminal ECs develop focal adhesions, a type of integrin that facilitates adhesion to ECM components [19, 20]. In general, ECs represent dynamic interfaces that respond to the CNS microenvironment and regulatory signals emanating from the blood and brain.

Although the endothelium is a critical component of the BBB, ECs cannot perform all BBB functions independently. Additionally, complex components of NVUs, such as ECs, glial cells, mural cells, and basement membrane (BM) /ECM components, can communicate to support and maintain the integrity of the BBB [21] [Fig. 1A]. In this case, pericytes (PCs) are embedded in the inner vascular BM lining on the abluminal surface of ECs, separating and connecting to ECs by gap junctions (connexin43) and direct peg-and-socket contacts (N-cadherin) that help maintain and stabilize the endothelium layer [22, 23]. In the outer parenchymal BM, the astrocytic endfeet almost completely envelops the vascular tube that connects to the other BBB cell types by gap junctions (connexin43 and connexin30) [24, 25]. Interestingly, astrocytes (ACs) are involved in ion and water regulation by showing enrichment of aquaporin 4, a regulatory water homeostasis protein in the CNS that regulates fluid flow throughout the CNS for clearance function [26]. In addition, ACs play a linkage function in the neurovascular coupling; ACs forward signals from neuronal activity to blood vessels for the regulation of cerebral blood flow [25]. The basal lamina, which includes the inner vascular BM (which connects ECs and PCs) and outer parenchymal BM (which extends from ACs to the vasculature), is another critical component of the BBB because it acts as an additional barrier and anchor for molecular signaling, of which the ECM is a major component [6, 27]. ECM is a significant component, including type IV collagen, laminin, nidogen, and other glycoproteins that offer an additional barrier and an anchor for molecular signaling across the barrier [27, 28]. These distinct components are well-organized to minimize the entry of unsafe molecules that contribute to a healthy CNS.

### Transportation pathways across BBB

Under physiological conditions, the BBB permits the supply of nutrients and regulates the influx of metabolites via both passive and active mechanisms [Fig. 1C]. The endothelial membrane contains a high concentration of zwitterionic phosphatidylcholine, phosphatidylethanolamine, and cholesterol, contributing to tight lipid packing [29]. On the lipid bilayer side, the motion of phospholipid tails creates transient gaps that allow for the passage of gas molecules (CO_2_ or O_2_) or a trace amount of water [30]. Nonetheless, membranes are associated with various embedded proteins, including intercellular junctions and transporters for mediating transportation [31]. At the intercellular cleft site, small molecules (< 500 Da) can cross the BBB via free diffusion (paracellular route) [32]. Large molecules can be transported via carrier-mediated, receptor-mediated, or absorption-mediated transport (the transcellular route) [15, 16, 33]. Amino acid transporters (e.g., LAT1/2, CAT1/3, SNAT1/2/3/5), carbohydrate transporters (e.g., GLUT1), monocarboxylic acid transporters (e.g., MCT1), fatty acid transporters (e.g., FATP1/4, MFSD2A), nucleotide transporters (e.g., CNT2, ENT1/2), and organic anion and cation transporters (e.g., OAT3, OATTP1A4, OCTN2, OCT1/2/3) are increasingly being considered as potential carriers of influx blood [15]. The receptor-mediated transcytosis pathway requires binding a receptor such as insulin, insulin-like growth factor, or leptin to transport macromolecules through membranous vesicular machinery such as lipid rafts or membranous caveolae. In contrast, absorption-mediated transcytosis refers to macromolecules transported through membranous vesicular machinery via positively charged molecular carriers with non-receptor binding [33, 34].

In contrast, brain-to-blood efflux clearance transporters perform in parallel with blood-to-brain influx transporters, helping to reduce the uptake of metabolites into the brain. The predominant efflux transporters have been identified, including the ATP-binding cassette (ABC) transporters, capable of limiting metabolite distribution to the brain and Aβ system, enabling the reintroduction of endogenous metabolites into the bloodstream [35, 36]. ABC transporters, including P-gp, BCRP, and MRP4, are located at the luminal membrane of EC, which prohibits the brain distribution of metabolites by pumping them out of ECs toward circulating blood [35]. On the abluminal side, the clearance system of Aβ involves lipoprotein receptors, which mediate Aβ toxin clearance, resulting in lower cerebral Aβ deposition [37]. In addition, cytochrome P450 (CYP-450), located on the endoplasmic reticulum membrane, is well expressed in the BBB and plays a role in the metabolism of medication to inhibit and induce drug interactions in brain cells [38]. The brain-to-blood system provides insights into eliminating endogenous metabolites and neurotoxic compounds from the CNS. However, numerous reports describing a deficiency or disruption of the brain-to-blood system suggest that it contributes to the progression of neurological pathology, although the molecular components and regulatory mechanisms involved remain unclear. Therefore, additional research is required to understand this mechanism fully.

### Pathological properties of BBB

In terms of the pathological process, accumulating evidence indicates that BBB dysfunction or BBB breakdown is one of the early pathophysiological hallmarks in neurodegenerative disease such as Alzheimer’s disease (AD), Parkinson’s disease (PD), Huntington’s disease, and amyotrophic lateral sclerosis [7, 39]. At the cellular level, the primary pathogenic feature is the migration of immune cells and mediators across the BBB. The disassembly of the intercellular junction allows for the entry of neurotoxic risk factors such as neurotoxic environmental factors, neurotoxic molecules into the brain, inflammatory factors, microbial pathogens, and immune cells [40], resulting in a variety of effects such as inflammatory and immune responses of glial cells to secrete neurotoxic cytokines (e.g., TNF-α, IL-1/6/12/18) and chemokines (e.g., CCL2, CXCR4), which contribute to the development of neurodegenerative disease [41]. Recent reports indicate that BBB breakdown is a risk factor for AD dementia, considered an early AD biomarker (Aβ deposition and phosphorylated tau) occurring before standard AD biomarkers. As a result, a permeable BBB creates unexpected hazards for neuronal cells.

At the molecular level, disruption of the BBB has been observed in several neurological diseases associated with genetic mutations. Genetic risk factors, such as mutations in ECs genes encoding TJ proteins, transporters, or ion channels (e.g., OCLN, SLC2A1, or MFSD2A) or external genetic factors (e.g., APOE E4, hypertension, or diabetes), may be influenced by BBB impairment, promoting the development of neuropathological disorders [3, 15]. For example, mutations in the coding sequence of a gene encoding tight junction endothelial protein, such as OCLN, which codes for occluding, or TJP1, which codes for ZO-1, results in the breakdown of the intercellular junction barrier, thereby impairing the function of the BBB and contributing to the pathogenesis of cortical malformation [42]. Another mutation in a gene encoding an influx/efflux system (e.g., transporters, carriers, or ion channels) results in the absence of transport metabolites, which prevent essential components (e.g., nutrients and ions) from reaching their destination. For instance, mutations in the SLC2A gene, which encodes GLUT1 as a remarkable transporter of glucose in ECs, result in glucose deficiency syndrome that impairs brain metabolism [43]. Therefore, genetic mutations affect specific cell types, resulting in BBB abnormalities unique to these cell types. Despite this evidence, a mechanism to clarify the pathway of neurodegenerative diseases related to BBB dysfunction remains unclear. Therefore, it is critical to conduct additional research to understand neurodegeneration better to develop therapeutic curves.

### Current limitation

The preceding arguments underscore the importance of developing a better understanding of neurological disorders to develop pharmacological targets or evaluate therapeutic agents crossing the BBB. Therefore, comprehensive in vitro, in vivo, and ex vivo studies should be conducted. In general, observations from animal and clinical experiments provide critical information on the physiology and pathophysiology of the BBB. However, understanding the cellular and molecular mechanisms is a significant challenge in these experiments. Although animal experimentation yields valuable results, ethical concerns have recently gained attention [44], and further incompatibilities between human and animal genomes cause several drug failures [45]. Consideration is given to the length of the experiment. On average, animal testing takes several months to a year, as do operational and consumable expenditures. Human in vitro BBB models are considered ideal for addressing limitations in animal investigations but require engineering methodologies that are both precise and cost-effective. Consequently, a strategic model that ensures physiological property similarity, cost-effectiveness, and operational speed is necessary.

## Advanced methodologies recapitulate the BBB

The invention of human mini-BBB models has broadened the possibilities and opportunities for investigating BBB pathophysiology and developing neuropharmacology. Recent BBB models closely resemble the microarchitecture of brain microvascular networks, making them an excellent tool for identifying potential therapeutic agents. This section discusses the current advances in the development of human mini-BBB models.

### Cellule types sources

As a building block for human brain reconstruction, brain NVUs cells are an exciting point of manipulation because they can be obtained directly from the postmortem brain or generated from stem cells [2, 46, 47]. Branch lines are sometimes referred to as primary or low-passage cells because they are isolated from brain arteries and display characteristics similar to the BBB in vivo, such as creating a substantial physical interface and expressing a range of functional markers during maturity [48]. However, this cell line is incapable of self-renewal, resulting in senescent functional and morphological alterations [49, 50]. To address the limitations of primary cells, immortalized brain cell lines have successfully immortalized primary cells while retaining BBB function and enabling cost-effective, simple expansion and long-term culture [51, 52]. However, models using immortalized EC lines, such as HUVEC, demonstrated a weakness in tightness after growth [53]. Owing to their many unique properties, human stem cells have recently been actively studied as a possible source for producing large numbers of capillary-like structures in the brain. In vitro BBB integrity reconstruction using human pluripotent stem cells showed a significant improvement over primary and immortalized cell lines [54]. For example, iPSC-derived brain microvascular ECs (iBMECs) and iPSC-derived brain capillary-like ECs (iBCLECs) exhibit similarities to in vivo BBB models in terms of gene and protein expression, including intercellular junctional complexes, influx/efflux transporters, and mechanosensor systems, indicating that BBB integrity and function are comparable to those observed in vivo [55, 56]. Another unique co-differentiation technique described by Lippmann et al. permits the production of endothelial and neural progenitor cells from hiPSCs, which exhibit the ability to reconstitute the native human brain microenvironment [57]. Notably, these cells have high transendothelial electrical resistance (TEER) and limited permeability to tracers, similar to the BBB characteristics seen in vivo [58]. In addition, mesenchymal stem cell (MSCs)-derived cells have recently shown similarities in vascular pericyte properties, enhancing the BBB model's physiological properties [59–61]. For example, Kim et al. have successfully demonstrated the contribution of MSC-derived pericytes in not only the formation of branching BBB-like microvasculature structures but also the enhancement of BBB tightness by showing the more robust of perivascular recruitment markers and reduction of permeability trackers [62]. These cell lines may be purified from the brain or derived from stem cells, thus providing representative values for in vitro BBB models. Although stem cells have shown their potential as in vivo models, other EC lines, such as primary EC and immortalized EC lines, are still widely used in research. Each cell line has both advantageous and detrimental properties; thus, it is critical to consider the features of the BBB when choosing a cell line for the study.

In the native human brain, various components of NVUs, including ECs, pericytes, glial cells, and mural cells, may interact to sustain and maintain the integrity of the BBB despite the fact that ECs are the core component of the BBB [6, 15, 63]. Therefore, ECs may be cultivated alone or in conjunction with other cell types. Depending on the number of cell types in the culture system, the in vitro BBB model can be categorized as either a “unicellular” or “multicellular” culture. The ‘unicellular culture’ paradigm is regarded as monoculture or single-cell culture, meaning that only one cell type, brain ECs, may develop. The ‘multicellular culture’ approach is also known as a co-culture or tri-culture model since it employs many cell types, as its name suggests. In this instance, brain ECs, together with other cell types, such as pericytes, astrocytes, smooth cells, and neurons, are grown. Recent research indicates that the ‘multicellular culture’ model more closely resembles the anatomic structure of the BBB by emphasizing the involvement of multiple cell types in BBB integrity [47, 49, 64, 65]. Thus, altering cell types in a culture setting is a promising strategy for in vitro BBB reconstructions.

### ECM sources

ECM composition can potentially impact the development of brain disease [66, 67]. Therefore, bio-components used in BBB building tend to recapture the brain microenvironment to correspond with endogenous signals in the cells [13]. These biomaterials with responsive characteristics may interact with cells through their physio- and bio-chemical properties, modulating the structural response of the in vitro BBB system [68]. For example, the biophysical properties of the hydrogel, such as stiffness, elasticity, topography, and degradation, may alter the microenvironment, influencing intracellular or intercellular signals, such as cell confluence or an inflammatory response [28, 69]. Thus, the physio- and bio-chemical characteristics of biomaterials should be adjusted to enhance cell adhesion and spreading while retaining their distinctive structures. Biomaterials used in BBB culture are often hydrogels obtained naturally from animals or manufactured in such a manner that they can mimic the microenvironment by transmitting complex ECM signals while providing the mechanical conditions essential for in vitro BBB restoration [70, 71].

Natural hydrogels derived from animals have improved intrinsic capacity and tissue structure [70, 72]. Collagen, laminins, alginate, elastin, hyaluronic acid, gelatin, matrigel, and other hydrogel-based materials extracted from bovine, mouse, and rat subjects have been widely documented to enhance cell adhesion in preparation for the creation of an endothelial monolayer [73, 74]. At the same time, collagen type IV and laminin are the most often used hydrogels in BBB cell–matrix adhesion and formation due to their interaction with focal adhesions and resulting in more compact packing [75, 76]. Matrigel is the chosen medium for 3D BBB self-organization because of its thick structure and weakly cross-linked gels, which promote cell migration and assembly [77, 78]. However, natural hydrogels have several disadvantages, including animal-derived components, batch-to-batch variability, and rapid disintegration [79]. Therefore, further research is required on ECM biomaterials suitable for human cells.

On the other hand, synthetic hydrogels or polymer-based provide another well-defined three-dimensional environment for BBB construction in vitro [80, 81]. Synthetic hydrogels generally consist of a polymer mesh with a water content that is mechanically and biochemically modifiable to preserve their structural integrity and elasticity. For instance, Pellowe et al. published an effective procedure for manipulating the mechanical characteristics of synthetic polyethylene glycol (PEG) hydrogels for cultivating endothelial-epithelial bilayers in the formation of BM-like structures [82]. Although synthetic PEG-based hydrogels exhibit excellent performance in architectural modification, PEG hydrogels alone cannot provide an ideal environment for cell adhesion and tissue formation [83]. PEG hydrogels can be modified to incorporate bioactive chemicals or natural polymers to increase their efficacy in human ECM-mimetic applications, and bioactive substances may be introduced into synthetic matrices to encourage cell growth or differentiation. For example, laminin combined with a modified PEG polymer enhanced iBMEC barrier formation [84]. However, varying degrees of cross-linking may affect physiological properties such as BBB integrity, which substantially influences the stiffness, viscoelasticity, and degradability of hydrogels [85]. For instance, hydrogel-decoded fibronectin and laminin result in very different BBB formation outcomes [73, 84]. Because varied hydrogels stimulate distinct biological responses, they must be modified to imitate a more realistic ECM microenvironment. Consequently, continuous ECM-mimicking attempts are being conducted to generate a mechanical environment that is ideal for in vitro BBB regeneration.

### In vitro BBB engineering strategies

**Fig. 2:**
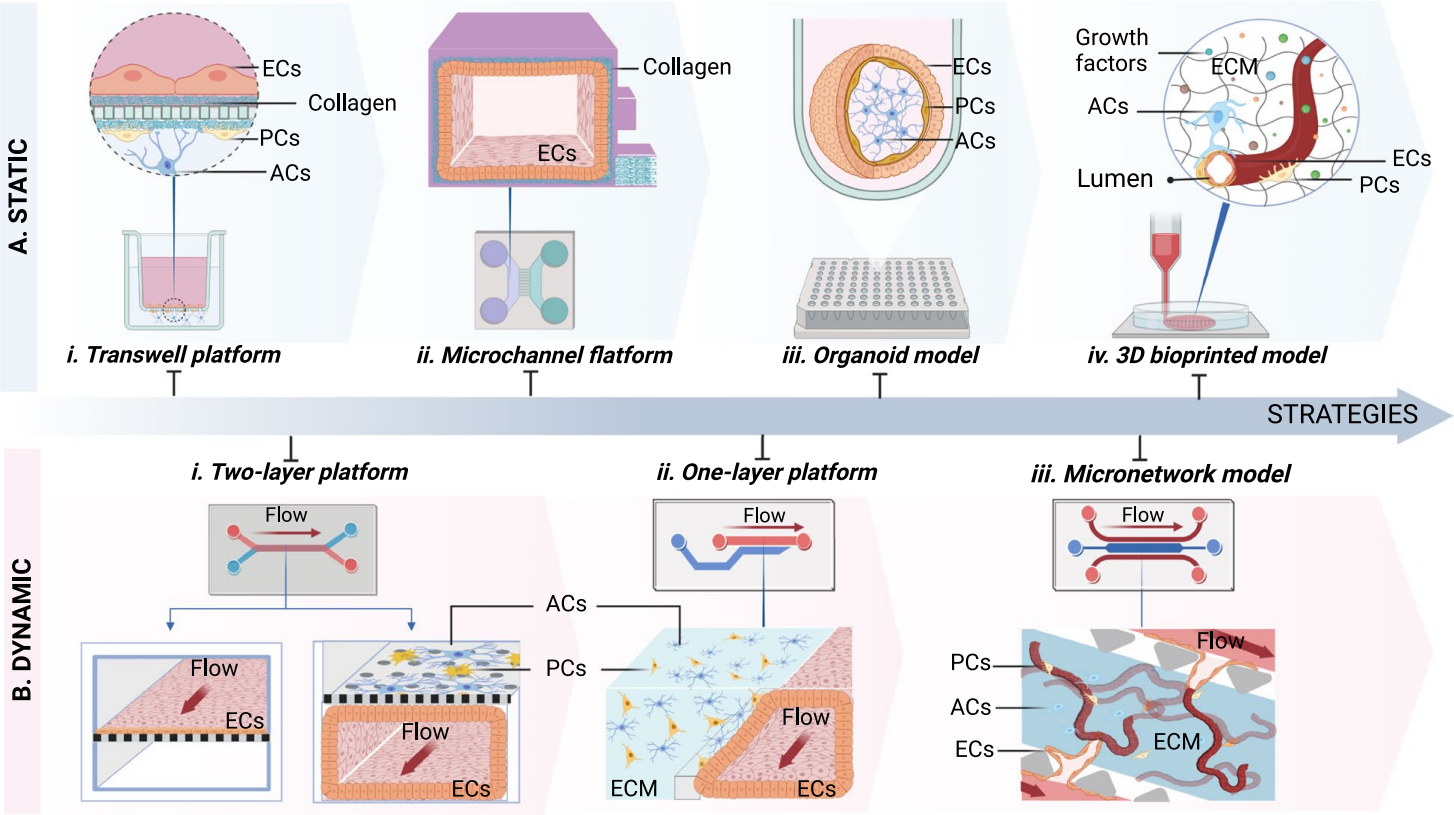
Evolution in creating in vitro BBB models. **A**. Model of the static BBB shows no fluid flow. i. The first generation of in vitro BBB models used a static endothelium monolayer separated by a porous membrane from co-culture pericytes and astrocytes called the transwell platform. ii. Microfluidics horizontal models with two main channels coupled by several migration channels enable the creation of a 3D ECs monolayer in a single compartment. iii. 3D NVU spheroid development in 96 well-plates. iv. The construction of three-dimensional lumen structures using 3D bioprinting methods. **B**. Model of the dynamic BBB allowing continuous flow generation in culture by a pumping device. i. Two-layer (vertical) designs similar to the transwell system, including ECs may form 2D or 3D EC monolayers in one compartment. At the same time, other cell types are cultured in a porous membrane-separated compartment. ii. A one-layer platform including a horizontal phage-based platform that utilizes the surface tension of ECM materials to separate 3D endothelial and other neurovascular unit cells. iii. A network model of the microvasculature using angiogenesis or vasculogenesis features may simulate the brain microvessel network on-a-chip. ECs represents endothelial cells; PC, pericytes; ACs, astrocytes

In vitro BBB models are critical and straightforward tools for various scientific applications, including investigating biological mechanisms, identifying therapeutic agents in neuroscience, and high throughput drug screening [10, 86]. Recent attempts to develop in vitro BBB models have yielded unexpected results, including forming a tight layer in a two-dimensional monolayer, a two-dimensional tubular structure, and even a complex microvascular system [87]. Indeed, the BBB is a dynamic interface that serves as a barrier between the bloodstream and neuronal environment and is susceptible to shear stress induced by blood flow [88]. Recent advancements in human in vitro BBB models have also captured this feature by introducing fluid flow to simulate dynamic blood conditions. Thus, in vitro BBB models may be characterized as ‘static’ or ‘dynamic’ according to their fundamental fluid flow/shear stress application characteristics [Fig. 2]. Static models maintain cells in the medium without applying flow or perfusion, allowing only minor environmental changes. In contrast, dynamic models maintain continuous flow in their culture using a pumping system to generate a specific pressure and flow rate, allowing for significant changes in the environment [89]. In vitro BBB models, in particular, demonstrate the critical role of fluid flow/shear stress in maintaining the BBB phenotype. Elbakary et al. showed a significant difference in response to shear stress between static and dynamic BBB models by boosting cell survival, barrier integrity through higher TEER values, and cell–cell connection [90]. Thus, dynamic culture models are significant because they can accurately represent human BBB.

#### BBB reconstruction in the transwell platform

The transwell platform is a typical platform of apical chambers separated by a porous membrane, and a multiwell plate format (basolateral chamber) is used to reconstruct the human BBB model [91] [Fig. 2A i]. Brain ECs have traditionally grown as a monolayer in an ECM-coated apical chamber. In contrast, other NVU cell types (e.g., pericytes, astrocytes, and/or neurons) have been cultured in basolateral chambers to simulate a section of the brain microvessel environment [91]. When fully developed, the apical chamber is the blood side, and the basolateral chamber is the brain side. To further illustrate the penetration and transport processes, the culture medium was perfused through the endothelium layer to replicate BBB perfusion. The equipment used in the transwell system permits the association of multiple cell types while maintaining a cost-effective and straightforward manufacturing process [12, 91]. Additionally, the transwell system with vertical diffusion is ideal for medicinal drug screening and delivery. However, these systems also have several limitations. For instance, a porous membrane may reduce the permeability of the tracker and hence limit cell–cell communication. Furthermore, this system cannot create three-dimensional structures that are similar to those observed in the human brain.

#### Reengineering BBB in the microfluidics

The first microfluidic BBB device was dubbed an organ-on-a-chip. Similar to the transwell concept, the BBB-on-chip is a two-layer device formed of two parallel hollow channels separated by a porous membrane [92, 93] [Fig. 2B ii]. The two hollow channels were adjusted to enable the formation of a monolayer of brain ECs in either a planar or tubular configuration. The brain monolayer endothelium is formed on one side of the ECM-coated porous membrane, whereas the other side is left vacant or ornamented with different NVU cell types. The controlled pumping system generates the flow of culture media via the brain endothelial channel, simulating the dynamic features of the natural microvessel [93]. Perfusion across the BBB layer was used to assess the integrity of the BBB, thereby producing an artificial brain model. For example, Park et al. introduced a microfluidic organ-on-a-chip BBB model that incorporated a monolayer tubular endothelium structure forming a vascular channel and human brain PCs and ACs on the upper surface of a porous membrane, recapitulating the native human BBB with a functional barrier [94]. Owing to its strong resemblance to genuine BBB barrier functions, it is recognized as a valuable tool for examining neurological diseases and drug screening. Despite considerable developments, the vertical architecture has some limitations, including the need for a porous membrane for cell laying, which may impede tracker permeability, restrict cell–cell contact, and impose extra layer connection procedures during operation.

Another microfluidic design subsequently demonstrated that single-layer structures are preferable to two-layer model structures in technology gap-filling. These systems consist of two or three major hollow channels on the same plane that are connected by several microchannels (a few micrometers in width) [Fig. 2A ii], a phage-based layer [Fig. 2B ii], or multiple pillars (gap width of a few micrometers) [Fig. 2B iii]. ECs were seeded onto an ECM-coated surface to create a monolayer or implanted inside the ECM hydrogel to generate a network. Cho et al. demonstrated two major channels separated by several microchannels. The ECs were covered to produce a tight tubular structure in the main channel; another main channel and microchannel were employed to check perfusion [95]. Another format developed by OrganoPlate creates a hollow through-curtain structure (dubbed phase guide) utilizing the surface tension of the ECM hydrogel. On one side of the phase guide, human brain PCs and ACs are implanted inside the ECM, whereas human brain microvascular ECs (TY10) are produced in tubular structures. On the other hand, culture media are perfused through the BBB layer for PCs and ACs development [96]. Additionally, the single-layer microfluidic platform has the potential for the formation of microvascular networks, in which ECs are seeded as monolayers on the surface of an ECM hydrogel, and another NVU is implanted inside the hydrogel to facilitate migration and self-assembly [97, 98]. When stimulated by angiogenesis or vasculogenesis growth factors, ECs sprout from one side of a horizontal microfluidic platform to the other. This sprouting process permits vessel branch expansion, culminating in network formation [99, 100]. Besides BBB design, microfluidic devices have shown their physiological relevance by using fluid flow in a tube or micro-network structure developed at the micrometer size to provide a realistic representation of BBB physiology.

#### Reconfiguration of BBB with organoids

Human BBB organoids are three-dimensional structures generated by microvascular cells' migration, proliferation, and self-organization, in which NVU cells may self-reconstruct a three-dimensional form [101, 102] [Fig. 2A iii]. Recently, significant progress has been achieved in finding BBB organoids that exhibit BBB characteristics, such as high quantities of intercellular junction proteins, transporters, and carrier proteins, for future applications in research on BBB drug transport and toxicity studies. Cho et al. demonstrated a tight solid model with outer surface ECs and PCs wrapped around an ACs core linked by intercellular connection proteins by utilizing compacts of human ECs, human PCs, and ACs under low ECM adherence conditions (ZO-1 and VE-cadherin) [64, 103]. Additionally, Nzou et al. described spheroids of human brain microvascular ECs (HBMECs), human PCs (HPs), human ACs (HAs), and human neuronal cells (HCN-2) [104]. Despite significant progress in developing BBB spheroid models, these models still have several limitations in depicting BBB activity [105]. For instance, the solid block in a spherical construction is a significant disadvantage in permeation monitors because of the difficulties in monitoring the internal core. Additionally, it imposes a size constraint on organoids while assessing their nutritional content, resulting in organoid fragmentation and survivability. Additionally, because the existing BBB organoid model acts in a static culture environment, the ECs interface does not experience natural fluid flow or shear stress. However, this may be adjusted by modifying the genotype and phenotype of the BBB. Consequently, the BBB spheroid model appears inappropriate for transportation studies.

#### 3D printing models mimicking the BBB

3D bioprinting is a method of printing a 3D structure from living cells and biomaterials (bio-ink) in which printers can deposit cells and scaffolds concurrently at precisely regulated 3D locations [106]. Recent advances in 3D printing and bioprinting have enabled the reconstruction of microvascular systems by stacking tissue-like structures using high-resolution ink or bio-ink [107–109] [Fig. 2A iv]. In tandem with the development of 3D bioprinting technology, efforts have been made to produce a structure comparable to the tubular structure of the microvasculature in the brain [74, 110, 111]. Lee et al. mimicked a vascular lumen structure by developing a perusable channel based on the liquefaction of gelatin and the gelation of collagen using a layer-by-layer bioprinting approach. The results demonstrated the barrier function of vascular channels by showing the expression of tight proteins (VE-Cad) and restriction of BSA/dextran-free diffusion [112]. Another strategy is to directly print the tubular blood vessel structure utilizing the co-axial bioprinting technology, in which the bioink within the core is enclosed by another bioink crosslinker exhibiting the configuration of hollow structures [113–116]. It is preferable that the inner nozzle is longer than the outer nozzle so that the crosslinker can flow along the outside surface of the inner nozzle [116–118]. Gao et al. successfully generated multiple-layered hollow conduits to reconstruct blood vessel-like tissues by using co-axial bioprinting technique. The hybrid bioink containing a mixture of vascular-tissue-derived decellularized extracellular matrix (vdECM), alginate, endothelial progenitor cells (EPCs), and atorvastatin/poly(lactic-co-glycolic acid) (PLGA) microspheres offers a conducive environment to promote the formation of vascularization. The core–shell nozzle is injected with a CaCl_2_ solution (CPF127) for ionically crosslinking by diffusing the Ca^2+^ to crosslink alginate in the hybrid bioink. The remarkable in this research is not only the construction of multi-layer hollow structures with a wide range of diameters by regulating the core–shell nozzle but also the creation of a perfusable and functional in vitro vascular model [119–121]. By applying this technique, Cho's study team attempted to reconstruct a human blood–brain barrier lumen dual-layered structure utilizing co-axial 3D bioprinting, in which PCs were embedded in the GelMA hydrogel in the shell structure. At the same time, the core component contained only human ECs in gelatin, as removing the gelatin during post-processing revealed a hollow cylindrical structure of microvessels with a high capacity for cell survival [122]. Although the characteristics of BBB growth have not been shown or validated, more research should be undertaken to obtain more relevant data. According to the results, bioprinting techniques are considered promising models for in vitro BBB maturation. Nevertheless, further studies are required to increase their capabilities. Consequently, BBB models generated by 3D printing and bioprinting still need further improvement. Once a 3D bioprinting model demonstrates BBB function, it is a feasible choice for high throughput in vitro models.

### Depiction of the human in vivo-like model

The functioning of the BBB enables the selective delivery of nutrients and growth factors to neuronal cells. It acts as a protective barrier, separating the blood from other brain tissues. While this has been demonstrated in in vivo models, conventional in vitro culture techniques require the maintenance of neuronal cells in a solution containing nutrition and growth factors in the absence of blood flow. To further elucidate BBB function in in vitro models, many studies have demonstrated high barrier resistance and the tracer permeation limitation stated in the TEER measure to evaluate barrier performance, decrease, and the permeability test [123, 124]. As barrier integrity exhibited, cell–cell interactions are demonstrated as specific protein localization [125]. Functional localization is observed for specific junctional proteins, and functional transporter proteins, including intercellular junction complexes, carriers, and transporters, are required for the BBB to operate properly [15]. For example, in an in vitro BBB model, the junctional localization of specific tight junction proteins (ZO-1/2/3, occludin, claudin-3, claudin-5), adherence junction proteins (PECAM-1, E-cadherin, N-cadherin, and VE-cadherin), and transporters (GLU-1 and transferrin) have been effectively established [126]. To further illustrate actual human BBB models, cellular gene expression in an in vitro model demonstrates the direction in which genes are typically expressed in the BBB in vivo [124]. Recently, Vatine et al. demonstrated a remarkable result in the development of a vertical microfluidic device using iBMECs to form a tightened tubular BBB monolayer by showing intercellular junctional complex proteins (e.g., ZO-1, occludin, PECAM-1), transporters (e.g., P-gp, BCRP, and MRPs), and mechanosensory proteins (e.g., caveolins), further enhancing BBB integrity by reducing molecule permeability (FITC-dextran at 3 kDa) while presenting a high resistance value (1,500 Ω.cm^2^ in TEER), demonstrating the critical necessity of a functional BBB in the protection of cytotoxicity from whole blood perfusion while nutrients are filtering for brain cell development [127]. As a consequence of the present certification, microfluidic BBB models integrating human stem cells display an increased resemblance to in vivo BBB structures, which play an increasingly important role in the discovery of neurological science for further research on elucidating the BBB response to internal and external stimuli.

## Biological findings from human mini-BBB models

Numerous studies published in the past several years have shown a relationship between BBB failure and neurodegenerative disease, indicating that BBB disruption is a risk factor for neurological disorders such as ischemic stroke [128], AD [129], and PD [130], among others. However, neurodegenerative disease linked to BBB disruption remain poorly understood in vivo. In contrast, in vitro BBB models have been recognized as potential tools for studying the biological mechanisms underlying neurodegenerative diseases. In this section, we discuss the improvements made in neurological disease modeling to explore the biological mechanism using the human mini-BBB model.

### Brain ischemia

**Fig. 3:**
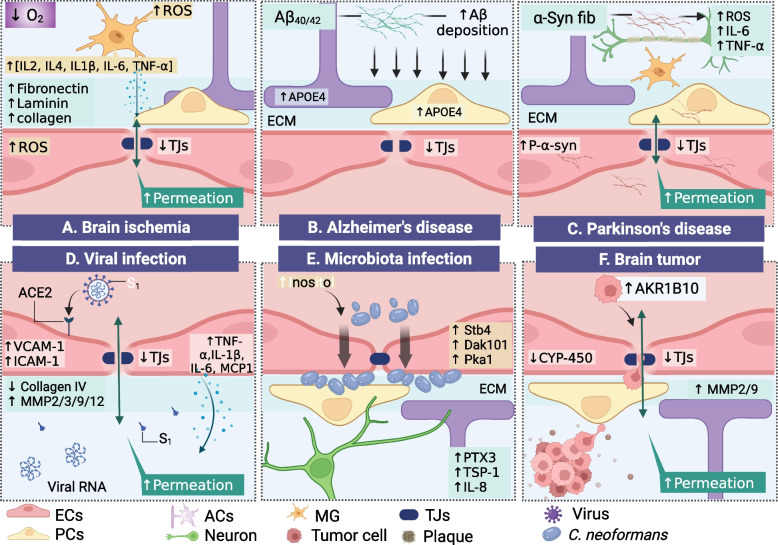
Human neurological disorders modeling. **A**. Human BBB model used in the study of hypoxic-ischemia, low oxygen condition-induced neuroinflammation, and BBB disruption. Human BBB model for assessing the stimulatory impact of **B**. Aβ clearance in Alzheimer’s disease. **C**. α-synuclein fibril in Parkinson’s disease. **D**. Human BBB model for elucidating the course of SARS-CoV-2 infection into the brain, exhibiting the effect of EC-induced ECM dysfunction and BBB disruption on the S1 spite protein binding to the AEC2 receptor. **E**. Use of the human BBB model to explore intestinal pathogenic fungal infection into the brain, indicating *Cryptococcosis neoformans* penetration and formation of clusters in the endothelium abluminal site. **F**. A physiological human blood–brain tumor barrier model for investigating of tumor cell intravasation. ECs represents endothelial cells; PC, pericytes; ACs, astrocytes; MG, microglia; TJs, tight junction

BBB disruption has been identified as a significant feature in preclinical models of ischemic stroke, facilitating the advancement of brain injury [128, 131, 132]. The presence of stroke reveals the initial increase in BBB permeability, which is thought to occur partly because of a disrupted junctional protein function field [131]. Following this initial breakdown, a neuroinflammatory reaction likely results in a continuous increase in permeability. The initial disruption of junctional protein expression results in increased BBB permeability, which cannot be explained entirely in preclinical data owing to observational limitations at the cellular and molecular levels. Otherwise, the clinical mimicking of stroke symptoms using a human BBB model convincingly demonstrates the course of harm. Recent findings in in vitro BBB models suggest that hypoxia-induced BBB malfunction has been linked to an increase in the expression of potentially damaging molecules such as vascular endothelial growth factor, hypoxia-induced factor 1-alpha (HIF-1-alpha), and heat shock proteins (HSP) [133]. Additionally, low oxygen conditions exhibit an antioxidant imbalance, which is linked to neuroinflammation through glial cell activation, resulting in pro-inflammatory cytokines and chemokines such as IL-2, MCP-1, IL-4, IL-1, TNF-α, and IL-6, in which pro-inflammatory factors dramatically influence the integrity of the BBB, resulting in high permeation. Another microfluidic BBB model demonstrated a leaky barrier under hypoglycemic and hypoxic conditions through mitochondrial malfunction, resulting in ATP deficiency [134] [Fig. 3A]. In contrast, hypoxic conditions enhance BBB functionality by boosting intracellular junctions (e.g., ZO-1, claudin, occludin, PECAM-1), transporters (e.g., GLUT-1 and AQP4), functioning efflux pumps (e.g., MDR-1, P-gp), and ECM components (e.g., collagen IV, laminin, perlecan, fibronectin, SPARC, and agrin) [94]. Thus, human BBB in vitro models is used under imitating conditions to establish physiological settings similar to those observed in stroke, elucidating the dominant influence of individual cell-induced BBB dysfunction and neurological disorders. However, the mechanisms of this disease have not yet been explained, and further evidence is needed.

### Alzheimer’s disease (AD)

Amyloid-beta (Aβ) deposition along the cerebral vasculature is a characteristic of AD pathogenesis, indicating BBB endothelial cell failure to balance Aβ levels in the brain and plasma [15]. Numerous studies in animal models have revealed that Aβ disrupts the BBB by interfering with its transit [129]. However, a recent publication asserted that mouse and human cells have significantly differential Aβ clearance across the BBB [135]. Therefore, a human brain model simulating the AD state is required to investigate the pathways of Aβ buildup in the brain and its clearance via the BBB layer. Blanchard et al. recently described a three-dimensional human microvascular network model comprising ECs, PCs, and ACs encapsulated in matrigel, demonstrating that PCs and ACs operate as primary sources of genetic risk factor (APOE4) expression, thereby enhancing Aβ accumulation [136]. Other human BBB models have been used to examine the Aβ_40/42_ uptake kinetics between the luminal and abluminal sides of the human endothelium layer, indicating that a high quantity of Aβ_42_ on the luminal side improves BBB uptake capacity and subsequently induces BBB failure [137]. Shin et al. demonstrated the dysfunction of barrier function in AD disease models by showing Aβ buildup on the abluminal side of the endothelium, indicating an increase in BBB permeability via downregulation of tight junction expression (claudin-1, claudin-5, and VE-cadherin) and upregulation of MMP2 and reactive oxygen species (ROS) production [138]. Additionally, pro-inflammatory factors such as IFN-ɣ, IL-1, IL-6, IL-8, and TNF-α generated by AD neuronal cells are related to increase in BBB permeability [Fig. 3B]. While numerous AD animal models have revealed early BBB disruption before the onset of AD pathology, the underlying mechanism remains unknown. Engineering-wise, the human BBB model displays the intricate physiological and pathological aspects of the BBB; nonetheless, the significance of the BBB in AD remains unknown. Thus, further applications of the in vitro BBB model to neurodegeneration should be constructed to understand the novel aspects of cell–cell interactions at the molecular level, which is a fundamental limitation of the in vivo model.

### Parkinson’s disease (PD)

Insoluble α-synuclein (α-syn) deposition has been implicated in the etiology of PD by promoting synaptic failure and neuronal death [139]. Numerous studies have shown that the accumulation and aggregation of α-syn may cause neuronal damage [130]. Additionally, specific strains of α-syn are hypothesized to facilitate the spread of pathogenic features inside the brain through migration between brain cells and across BBB interfaces [140]. However, in vivo models cannot simulate the interactions between α-syn and NVU cells. Pediaditakis et al. demonstrated a vascular neuronal interface to investigate the effect of α-syn fibrils on the NVU in a PD brain model. This model showed that α-syn fibrils induced phospho-Syn129 pathology in neurons, astrocytes, microglia, and especially in ECs, resulting in mitochondrial damage, increased intracellular ROS production, glial cell activation, and increased secretion of pro-inflammatory factors (IL-6 and TNF-α). Additionally, α-syn fibrils directly impair the BBB by disrupting tight junctions (ZO-1) and increasing inflammatory factors in ECs (ICAM-1) [141]. Remarkably, fundamental disease processes are uncovered using the human brain model [Fig. 3C].

### Virus infection

Although germ buildup in the human brain results in neurological disorders, the process by which viruses invade or affect the brain remains unclear. Recent research has examined the invasion process, concluding that the BBB serves as the primary entry point for the virus to the brain [142, 143]. Because individuals with COVID-19 exhibit various neurological symptoms, their neuropathology remains unknown [144, 145]; due to the urgency of this issue, the impact of SARS-CoV-2 on the brain must be investigated immediately to offer prompt remedies to the repercussions. Multiple researchers have attempted to rebuild human BBB models to demonstrate the effects of SARS-CoV-2 infection on the brain. For example, Buzhdygan et al. showed that SARS-CoV-2 affects the brain endothelium, resulting in the expression of ACE2 receptor on brain ECs to facilitate the capture of SARS-CoV-2 subunit S1 despite proteins, thereby triggering an increase in BBB permeability via loss of intracellular junctions (ZO-1), upregulation of factors mediating inflammatory processes in ECs (ICAM-1, VCAM-1), pro-inflammatory release factors (IL-1β, IL-6, CCL5, CXCL10), and elevated barrier integrity matrix metalloproteinases (MMP2, MMP3, MMP9, and MMP12) [146]. However, the disintegration of the BBB caused by SARS-CoV-2 is still debatable. Previous research has shown that SARS-CoV-2 can penetrate the BBB without changing intercellular connections. Krasemann et al. validated SARS-CoV-2 infection in BBB monolayer cultures, demonstrating that the ACE2 receptor is transported across the intact BBB [147]. Later, Zhang et al. claimed that the BBB was preserved through the SARS-CoV-2 cross-BBB model, but the damage occurred because of decreased collagen IV and increased MMP9 [148]. In summary, SARS-CoV-2 may cause BBB destruction by altering matrix metalloproteinases, initiating pro-inflammatory responses, and subsequently, these factors return to target the BBB. These findings corroborate those of the human BBB model by indicating that SARS-CoV-2 promotes barrier breakdown; hence, current models can address urgent concerns to identify a panacea during the pandemic [Fig. 3D].

### Microbiota infection

The human CNS infects the gut microbiota. In this scenario, the BBB is regarded as the primary entry site of intestinal pathogens and their metabolites [149, 150]. However, the influence of infection and entry routes through the BBB remains unclear. For this reason, a human mini-BBB or gut-brain model is needed to elucidate the effect of the pathogen on gut disorders and its subsequent impact on the brain. Kim et al. recently demonstrated a human brain-relevant on-a-chip that included a hollow BBB interconnected with a 3D matrix, with a tubular EC monolayer tubular structure lying next to implanted PCs, ACs, and neurons in the 3D hydrogel [151]. Fungal pathogens (*C. neoformans*) were introduced into the BBB lumen. Their attachment to the brain area was observed, showing host-derived neurotrophic factors (inositol, Stb4, Dak101, and Pka1) enhance *C. neoformans* recruitment through the BBB layer. Colonization underneath the EC layer is induced by the release of paracrine factors (PTX3, TSP-1, and IL-8), which may act as a neuroinflammation modulator [Fig. 3E]. Thus, human BBB models are advantageous for deciphering host signaling pathways responding to microorganism infection and monitoring BBB-microorganism interactions. BBB models will provide insight into hitherto unexplored pathways linking gut pathogen effects to the brain in the next several years.

### Brain tumor

It is widely known that vascularity deteriorates as brain tumors develop [152]. However, recent findings in brain tumor vessels indicate that NVUs are distinct from other peripheries. They contain an intact BBB, which inhibits therapeutic agent entry and confers resistance to chemotherapy but is not an impenetrable barrier to metastasizing cancer cell transmigration. Recent advances in human blood–brain tumor models have yielded extraordinary results. Deligne et al. revealed that chemotherapeutic resistance might propagate across the BBB layer in the presence of pediatric diffuse intrinsic pontine glioma cells, resulting in altered CYP expression and drug transport failure across the blood–brain tumor barrier [153]. Additionally, the microfluidic lung-BBB-brain model recapitulates lung cancer metastasis to the brain via the BBB system, indicating that tumor cell expression of Aldoketo reductase family 1 B10 (AKR1B10) is tightly linked to BBB extravasation across the BBB, resulting in the upregulation of MMP2 and MMP9, and altering the ECM, which may facilitate inflammation [154] [Fig. 3F]. Thus, understanding brain tumors at the cellular and molecular levels is critical for developing an effective treatment strategy. In contrast, the human blood–brain tumor barrier model provides an alternative brain tumor environment.

These revolutionary results pave the way for a plethora of new directions in fundamental brain research, indicating that a human mini-BBB model is a potential tool for neurological examination, acting as an imprinting template for pathogenic conformational alterations from the peripheral to CNS or from the CNS to the periphery. Based on these findings, human BBB models may be used directly to investigate the fundamental mechanisms underlying neurodegeneration.

## New tools for personalized medicine

### Models for drug delivery

**Fig. 4:**
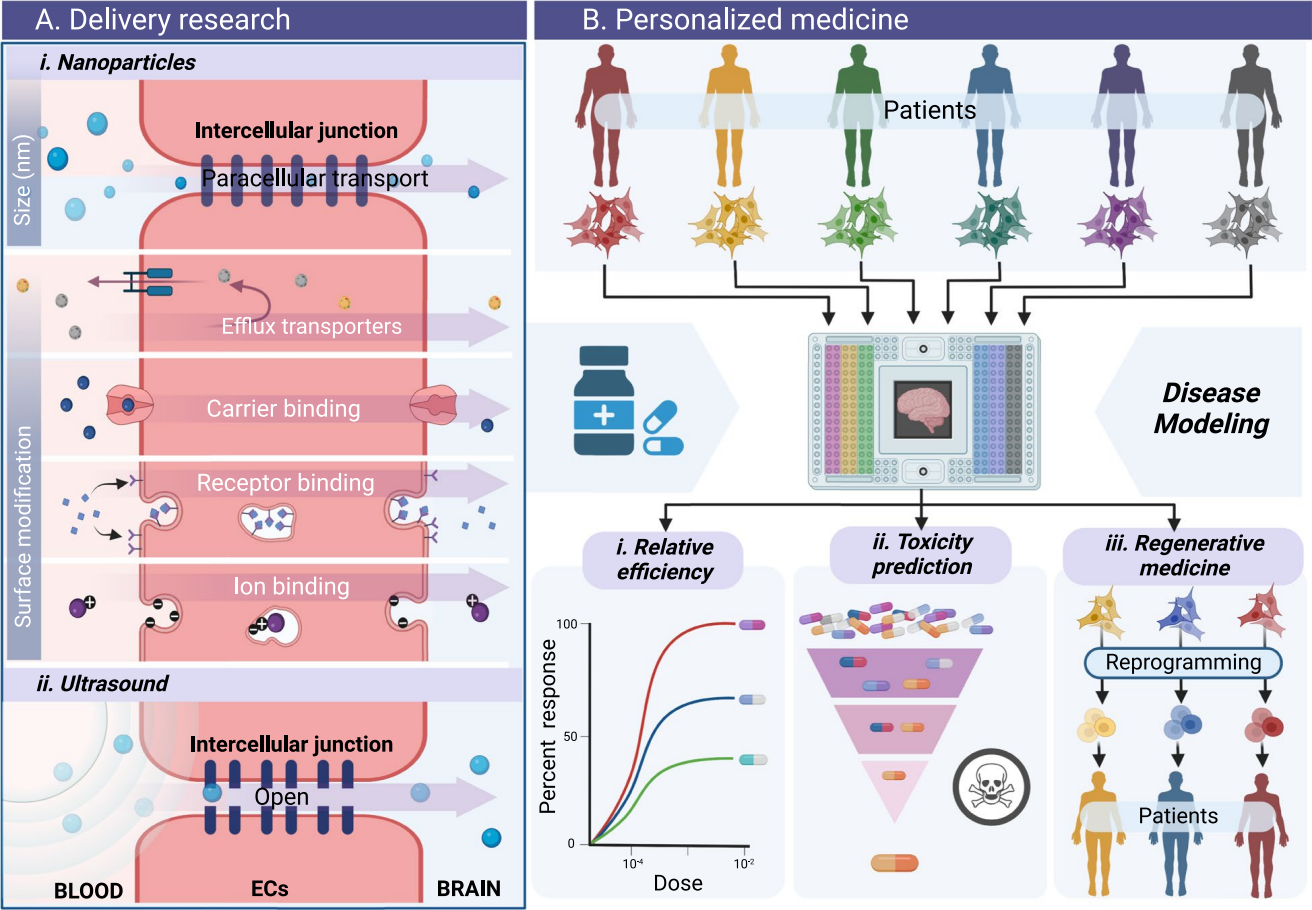
Human BBB models application in neuropharmacology. **A**. In vitro BBB models provide a straightforward method for studying the penetration of various types of pharmacological molecules. i. In vitro human BBB models for the research of tailored nanoparticle penetration. Nanoparticle-mediated drug delivery engineering techniques comprising size and surface modification are employed to functionalize the nanoparticles, such as ligand integration, amphiphilic encapsulation, or charge alteration for both transcellular and paracellular routes. ii. The in vivo-like structural models for research on the effect of ultrasound on BBB opening for drug delivery. **B**. In vitro models of the human BBB may also be utilized in place of animal models by employing patient cells in a high throughput manner for assessing i. drug efficacy and ii. safety in preclinical investigations, and iii. stem cell therapy as the next generation of medicine evolves. ECs represents endothelial cells

Although considered a necessary component of CNS function, the BBB presents an impenetrable barrier to therapeutic medicines [155]. The preceding points emphasize the crucial significance of expanding our understanding of neurological diseases to penetrate the BBB, specifically targeting a specific injured region [15]. As a result of our knowledge of the structure and function of the BBB, we now have a variety of drug delivery strategies to bypass this barrier and reach the wounded brain areas. The intact human BBB model enables the visualization of pharmaceutical distribution channels, which aids in medication effectiveness and conjugation into tailored nanoparticles (NPs)/ nanocarriers (NCs) [156] [Fig. 4A i]. The physical properties of NPs have a significant effect on their interaction with the brain endothelium, impacting their passage across the BBB [157, 158]. The affinity of hard and microscopic particles for the endothelium is greater than that of soft and large particles. A recent study indicated that the ability of NPs to permeate the BBB is governed by their physiochemical properties, including their size, shape, and surface properties. NPs with a diameter of up to 200 nm can penetrate the human BBB layer, while those with a diameter of less than 4 nm can pass through paracellular routes. Additionally, the surface of NPs is critical in the transcellular transport route, where the physicochemical properties associated with decorated NPs containing ECs carriers or transporters facilitate transport across the BBB [157, 159]. For example, transcytosis transportation channels are exemplified by high-density lipoprotein (HDL)-mimetic NPs containing apolipoprotein A1 or ionic surface modifications to enhance BBB penetration. Kumar et al. demonstrated the ability of rabies virus glycoprotein (RVG)-carrying small interfering ribonucleic acid (siRNA) transvascular delivery into brain cells, which opens a venue for siRNA encapsulation strategy, coated with RVG peptide as a targeting ligand for a CNS delivery model [160]. Another effective strategy for delivering therapeutic agents to targeted brain regions has been reported as exosome-endogenous nanovesicles, where the gene therapeutic agent siRNA was loaded into the RVG-surfaced modified exosome derived from immune cells. These engineered exosomes show specific transportation of siRNA into neurons, microglia, and oligodendrocytes in the brain for AD treatment [161]. As previously demonstrated, in vitro BBB models are advantageous for assessing the effectiveness of neuronal drugs.

Currently, high-intensity focused ultrasound (FUS) is deemed safe for opening the BBB to treat various neuronal disorders, most notably cancer [162, 163]. As a result, ultrasonography has emerged as a viable tool for optimizing drug delivery to the brain. Although various animal models have been developed, the specific mechanism by which FUS induces BBB opening and reversal remains unclear, necessitating further studies on drug delivery targets and side effects of cell damage [164]. A synthetic intact human BBB model provides an unbiased assessment of the physical influence on the BBB [Fig. 4A ii]. Recent research indicates that microbubbles generated by low-intensity focused ultrasonic waves alter the permeability of the BBB by mechanically disrupting tight BBB connections, thereby facilitating drug penetration into the abluminal side [165]. Another study revealed that therapeutic Alzheimer’s antibodies could be successfully delivered across the human BBB using FUS without inducing cell death or inflammation [166]. This minimally invasive approach, which may be repeated numerous times, temporarily disrupts the BBB and enhances the efficacy of medicinal drugs.

### Models for drug screening

Although numerous strategies for improving medication distribution across the BBB have been developed, the toxicological qualities of medicinal pharmaceuticals provide a barrier that must be overcome before commencing clinical trials [167]. Therefore, toxicity testing is crucial for novel drugs and conjugating agents for laboratory-produced NPs [168, 169]. Numerous scientific investigations have shown that novel drugs cause cytotoxicity and neuroinflammation, primarily by disrupting tight junctions, resulting in cell death [170, 171]. For example, Leite et al. found that Au-NPs and PLA-NPs triggered apoptosis, changed the BBB architecture, activated microglia, and increased cell stress and mortality through mitochondrial malfunction [172]. Owing to the advantages of in vitro cultures, an increasing number of studies have employed various models to investigate the neurotoxicity of medicines; consequently, in vitro brain models are frequently used during the early stages of developing novel neurological medications [86]. Several investigations have been conducted using standard well plate layouts to facilitate high throughput experiments and interoperability with standard laboratory equipment. The OrganoPlate (MIMETAS) [86], vascularized micro-organ (4 Design Biosciences) [173], BBB parallel array [174], BBB model [175], and multi-channel microfluidic device are microfluidic devices with a well plate interface. Therefore, in vitro BBB models are considered high-throughput screening tools for preclinical research [Fig. 4B ii].

### Models for regenerative medicine

Although animal research yields significant findings, the incompatibility between human and animal genomes contributes to the failure of many medications [44, 45]. Recently, patient-derived cells, particularly induced pluripotent stem cells (iPSC) and mesenchymal stem cells (MSC), have created an ex vivo system that may be used instead of standard preclinical approaches and serve as a model for personalized medicine [176–178]. Additionally, a study revealed that each individual has a distinct genotype and phenotype, implying that pharmaceutical responses are highly variable [179]. The emergence of body-on-a-chip technology, especially the coupling of organs to the brain using patient-derived cells, opens the way for personalized medicine by providing a straightforward tool for categorizing individuals according to disease or treatment response [127, 180]. Although there have been few studies on using iPSCs produced by patients, advancements in the human BBB model hold great promise. A recent study established the use of an in vitro BBB model to identify stem cell candidates for infiltration into ischemic stroke scenarios, thereby proving the efficacy of stem cell treatment for neuronal recovery after stroke [181]. In addition, clinical trials with MSCs patients in several types of neurological disorders (e.g., Amyotrophic Lateral Sclerosis, Stroke, and Traumatic Brain Injury) are underway; however, the MSC product release still controversial [178, 182]. Thus, human brain models with complete physiological characteristics replicate the intricate structure of the human microvasculature while integrating it with other organs by integrating with cells derived from the patient will demonstrate it in a specific disease, thus showing the promise of next-generation medicine in terms of personalized medicine [Fig. 4B iii]. We believe that by merging patient-derived cells with bio-fabrication for the replication of patients, we might make considerable progress in tailored treatment for neurological disorders.

Despite its many triumphs in fundamental research on a laboratory scale, the human brain model continues to meet obstacles in the pharmaceutical pipeline as a substitute for animal models in preclinical research. Currently, there are no definite and well-defined criteria for developing a specific human brain model by competent authorities, which means that progress toward animal-free models needs to be faster. However, the good news is that authorities have recently gained prominence in ethical issues surrounding the use of animal models in experiments, which means that once standards are established, the artificial human brain model will be an ideal substitute. To do this, specific and persuasive evidence demonstrating the superiority of human mini-brain models over particular animal models is required.

## Concluding remarks and perspectives

As advances described above, the human BBB is a unique vascular barrier formed by microvascular NVU cells lining the cerebral capillaries that regulate the transport of molecules into brain tissues. Mini-BBB models are artificial frameworks that mimic the human BBB for culture systems that can open up a new avenue for investigating neurological pathways and evaluating the effectiveness of biopharmaceuticals and medicines [183]. Human mini-BBB models that replicate CNS systems in combination with human cells and ECM components create a more relevant milieu, resulting in more meaningful results. The pathophysiological properties of mini-BBB models are becoming more apparent at the molecular and cellular levels, indicating potential biomarkers for drug developers. Second, mini-BBB models with structural modifications represent the native BBB genotype and phenotype. Therefore, the direct assessment of medicinal drug penetration using human BBB models is believed to be more efficient than in vivo models because it elucidates the transport mechanism. Finally, and perhaps most importantly, novel medicines must undergo a thorough toxicological assessment to guarantee their safety and cellular response. The next generation of medicines will be achieved shortly using a human cell model similar to the patient model. Although current versions of the human mini-BBB have shown considerable promise for broad use, certain limitations prevent them from serving as a substitute for in vivo models. The primary challenge is that the human BBB is a complex system with numerous hidden mechanisms that current technologies cannot fully illuminate. In contrast, the human BBB model is a simplified model that cannot fully enumerate the complex aspects of physiopathology, frequently necessitating confirmation using animal models. The secondary challenge is researching neurological diseases, toxicity, and medication response, which requires a long time to verify cell-to-cell interactions and guarantee the safety of novel therapeutic agents, while published human BBB models only survive for a few days. Owing to these challenges, further research on complex long-term BBB models in vitro is needed.

Although a complexity model is required, a suitable model for the intended application must be developed because complexity models are related to cost-effectiveness, operation control, and throughput. Integrating the BBB with additional cell types linked to disease scenarios may enhance the complexity of the BBB system. For instance, investigations of the BBB reaction to external stimuli require a simple BBB monolayer, whereas brain cancer development requires a sophisticated microvascular network system. As a result, the protocol and validation process must be tailored to the applications in question.

In addition, the advancement of the human brain model has expanded the possibilities for creating medicines, particularly CNS therapeutic agents. Recent advances in artificial human brain models have brought them closer to the microarchitecture of brain physiopathogenesis, serving as a potential tool for neurological research in neurodegenerative diseases and establishing them as attractive tools for basic drug discovery research. By gaining a better understanding of BBB physiopathology, the development of neuropharmacological drugs will become more accessible. Additionally, rebuilding the brain microvasculature and microvascular environments will be simpler than ever to undertake pathophysiological brain research, medication screening for delivery, toxicological analysis, and personalized medicine by providing easy tools and replacing animal studies.

In this review, we primarily address the present state and difficulties of mini-brain models for fundamental neuroscience research, neurodegenerative diseases, and neurological drug delivery. Although artificial mini-brain models have attained many physiopathological characteristics, they require further technical development to obtain native human BBB and brain properties. Thus, advancements in brain tissue engineering, biomaterial engineering, and microengineering are being made devoid of animal components for humans that are physiologically relevant under a micro-scalable controlled design. The discussion in this article will illuminate several alternative strategies for creating human BBB models. Numerous BBB models have been discussed in this review, and it is conceivable to connect them with other organs, such as the intestine for the gut-brain axis or the respiratory system, to replicate neurological abnormalities induced by external causes.

## Data Availability

Data sharing is not applicable to this article as no datasets were generated or analyzed during the current study.

## Abbreviations

2D: Two-dimensional
3D: Three-dimensional
ABC: ATP-binding Cassette
ACE2: Angiotensin Converting Enzyme 2
ACs: Astrocytes
AD: Alzheimer’s Disease
AKR1B10: Aldoketo Reductase Family 1 B10
AQP4: Aquaporin-4
Au-NP: Gold nanoparticles
Aβ: Amyloid-beta
AJs: Adherens Junctions
BBB: Blood–Brain Barrier
BM: Basement Membrane
*C. neoformans*: *Cryptococcus neoformans*
CAT1/3: Cationic Amino Acid Transporter 1/3
CCL5: Chemokine (C–C motif) Ligand 5
CNS: Central Nervous System
CNT2: Concentrative Nucleoside Cotransporter
COVID-19: Coronavirus Disease 2019
CXCL10: C-X-C Motif Chemokine Ligand 10
CYP: Cytochromes P450
E-cadherin: Epithelial Cadherin
ECM: Extracellular Matrix
ECs: Endothelial Cells
ENT1/2: Equilibrative Nucleoside Transporter 1/2
ER: Endoplasmic Reticulum
FATP1/4: The Fatty Acid Transport Protein 1/4
FITC-dextran: Fluorescein Isothiocyanate Dextran
FUS: Focused Ultrasound
GLU-1: Glucose Transporter 1
GJs: Gap Junctions
HDL: High-Density Lipoprotein
HUVEC: Human Umbilical Vein Endothelial Cell
iBCLECs: IPSC-derived Brain Capillary-Like Endothelical Cells
iBMECs: IPSC-derived Brain Microvascular Endothelical Cells
ICAM-1: Intercellular Adhesion Molecule 1
IFN-ɣ: Interferon gamma
IL-1β/2/4/6/8: Interleukin 1β/2/4/6/8
IJs: Intercellular Junctions
iPSC: Pluripotent Stem Cells
LAT1/2: Large Neutral Amino Acid transporter 1/2
LIFU: Low Intensity Focused Ultrasonic
MCP-1: Monocyte Chemoattractant Protein-1
MDR-1: Multidrug Resistance Protein 1
MFSD2A: Major Facilitator Superfamily Domain containing 2A
MMP-2/3/9/12: Matrix Metalloproteinase-2/3/9/12
MSCs: Mesenchymal Stem Cells
N-cadherin: Neural Cadherin
NCs: Nanocarriers
NPs: Nanoparticles
NVUs: Neurovascular units
OAT3: Organic Anion Transporters 1 and 3
OCT1/2/3: Organic Cation Transporter 1/2/3
OCTN2: Carnitine Transporter
PCs: Pericytes
PD: Parkinson’s Disease
PECAM-1: Platelet Endothelial Cell Adhesion Molecule 1
PEG: Polyethylene Glycol
P-gp: Permeability Glycoprotein
PLA-NP: Poly-Lactic Acid Nanoparticles
PTX3: Pentraxin 3
P-α-syn: Phosphorylated alpha synuclein
SARS-CoV-2: Severe Acute Respiratory Syndrome Coronavirus 2
SNAT1/2/3/5: Sodium-coupled Neutral Amino Acid Transporter 1/2/3/5
TEER: Transendothelial Electrical Resistance
TNF-α: Tumour Necrosis Factor alpha
TJs: Tight Junctions
TSP-1: Thrombospondin-1 (TSP-1)
VCAM-1: Vascular Cell Adhesion Molecule 1
VE-cadherin: Vascular Endothelial Cadherin
VEGF: Vascular Endothelial Growth Factor
WHO: World Health Organization
ZO-1/2/3: Zonula Occludens proteins 1/2/3
α-syn: α-synuclein
